# Study on the Pathogenic Mechanism of *Pasteurella multocida*

**DOI:** 10.3390/pathogens15090881

**Published:** 2026-08-22

**Authors:** Min Qiu, Jinru Zou, Donghua Zheng, Miao Song, Dan Yang, Zheng Yang, Rui Wang

**Affiliations:** 1 College of Veterinary Medicine, Inner Mongolia Agricultural University, Hohhot 010018, China; 102006095@btmc.edu.cn; 2 College of Pharmacy, Baotou Medical College, Baotou 014040, China; 13568619197@163.com (J.Z.); btyxyzdh@btmc.edu.cn (D.Z.); tinasongmiao@163.com (M.S.); yd102011031@163.com (D.Y.); 3 College of Pharmacy, Jinan University, Guangzhou 511443, China; 4 The First Affiliated Hospital of Baotou Medical College, Baotou 014010, China; 202006100@btmc.edu.cn

**Keywords:** *Pasteurella multocida*, virulence factor, pathogenesis, inflammasome

## Abstract

*Pasteurella multocida* is highly prevalent in animal populations. It is usually a component of the normal microbiome of the oral, nasopharyngeal and upper respiratory tracts of animals and can cause varying degrees of infection and even death in animals. *P. multocida* poses a threat to public health safety because humans can be infected by being bitten by infected animals or coming into contact with their nasal secretions. This article summarises the morphological and culture characteristics of *P. multocida*, in addition to serotyping, virulence factors and pathogenesis, aiming to explore the mechanism by which the pathogen establishes acute and chronic infections. The article also summarises the mechanism underlying the activation of inflammasomes after *P. multocida* infection, providing a reference for exploring the epidemiological laws and pathogenesis of the bacterium and for formulating prevention and control strategies.

## 1. Introduction

**Figure 1:**
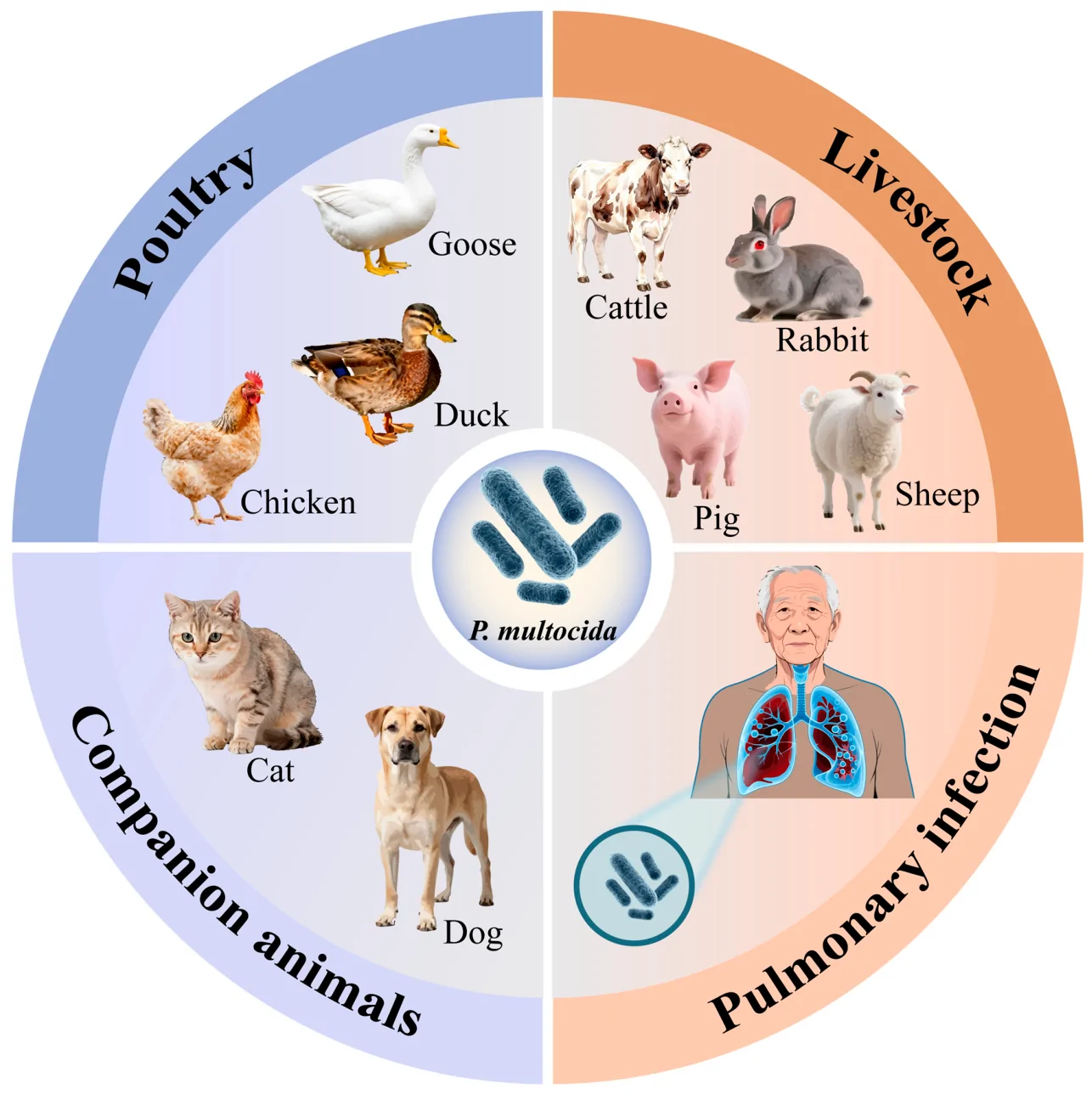
Host range of *Pasteurella multocida*.

*Pasteurella multocida* is one of the most common commensal and opportunistic pathogens found in domestic and wild animals worldwide and is highly prevalent in animal populations. It is often found as part of the microbial community in the oral, nasopharyngeal and upper respiratory tracts of animals [1]. *P. multocida* can cause serious infectious diseases in livestock, including bovine respiratory disease (BRD) [2], bovine haemorrhagic septicaemia and progressive atrophic rhinitis in pigs and fowl cholera, rabbit snuffles and pneumonia in small ruminants and pigs [3,4] (Figure 1). These diseases often have acute, febrile and highly contagious characteristics, causing considerable economic losses to the livestock industry. Furthermore, *P. multocida* can interact with other pathogens, contributing to the formation of respiratory disease complexes or endemic pneumonia in cattle and pigs [5]. *P. multocida* can be transmitted to humans through animal bites or contact with infected animals or carcasses, causing pulmonary infections, arthritis, encephalitis and other serious diseases [6,7,8].

Cats and dogs, as the most common companion animals worldwide, are the primary reservoirs and sources of *P. multocida* infection for humans [9,10]. The bacterium is a commensal of the oral and nasopharyngeal cavities of these animals, with carriage rates of 70–90% in cats and 20–50% in dogs [10,11,12]. Consequently, *P. multocida* can be isolated from approximately 75% and 50% of cat and dog bite wounds, respectively [10,13]. A recent retrospective study of 482 human cases further confirmed that animal exposure was the primary source of infection, with cats accounting for 54.1% and dogs for 29% of cases [10]. Zoonotic pathogens are usually transmitted to humans through animal bites or nasal secretions, and *P. multocida* is the most commonly isolated bacterium in human infections [14,15,16].

In recent years, rapid developments in molecular biology and immunology have enhanced our understanding of the pathogenic mechanism of *P. multocida* [17]. The pathogenicity of *P. multocida* is closely related to its complex virulence factors, including capsules, lipopolysaccharides (LPSs), outer membrane proteins, iron carriers, sialidases and toxins, which play a crucial role in bacterial colonisation, evasion of host immunity, invasion of cells and tissue damage [18,19]. Moreover, *P. multocida* infection is not a simple interaction between bacteria and host cells; rather, it involves complex host immune responses [20]. The activation of inflammasomes is one of the important mechanisms by which a host responds to *P. multocida* infection. Inflammasomes are multi-protein complexes that can rapidly assemble and activate in response to pathogen-associated molecular patterns (PAMPs) or damage-associated molecular patterns (DAMPs), triggering a series of inflammatory responses, which include the release of pro-inflammatory cytokines and pyroptosis in cells [21,22]. These responses help clear pathogens but may lead to tissue damage and the progression of diseases.

Although previous reviews have comprehensively summarised the virulence factors, epidemiology and clinical manifestations of *P. multocida* infections, a systematic overview of the host inflammatory response, particularly the role of inflammasome activation in this bacterium’s pathogenesis, remains lacking. Most existing reviews have focused on the bacterial side of the host–pathogen interaction, with limited attention to how the host immune system recognises and responds to *P. multocida* infection at the molecular level. The present review fills this gap by providing a focused and up-to-date synthesis of the molecular mechanisms by which *P. multocida* virulence factors trigger inflammasome activation and how this inflammatory response contributes to both bacterial clearance and tissue pathology. By integrating recent findings from in vitro and in vivo studies, this review presents a novel perspective that bridges bacterial pathogenesis and host immunology and may inform future research directions and therapeutic strategies.

The aim of this review is to explore the pathogenic mechanism of *P. multocida*, especially its relationship with inflammasome activation. By elucidating how *P. multocida* evades host immunity, invades cells and causes tissue damage through its virulence factors, as well as how the host responds to infection via inflammasome activation, we hope to provide new insights and strategies for the prevention, diagnosis and treatment of *P. multocida*-related diseases. This review will help us better understand the role of inflammasomes in anti-infection immunity, laying the foundation for the development of novel immunotherapies and vaccines.

## 2. Morphology and Serotypes of *Pasteurella multocida*

*P. multocida* is a small, rod-shaped or short bacillus with blunt ends and a nearly elliptical shape, measuring approximately 0.2–0.4 μm in width and 0.5–2.5 μm in length. It is Gram-negative and has no spores or flagella [23]. Infected diseased tissues or body fluids stained with Wright’s stain or methylene blue show typical bipolar staining. Freshly isolated virulent strains possess a mucoid capsule, which rapidly disappears upon cultivation. *P. multocida* is not highly fastidious and grows well on common media such as nutrient agar and blood agar, forming small circular colonies within 24 h at 37 °C; *P. multocida* does not grow on MacConkey agar, a feature that aids in its differentiation from other Enterobacteriaceae. When cultivated on blood agar plates for 24 h, it forms small, pale greyish-white, smooth and shiny dewdrop-like colonies with neat edges without haemolysis. It is an aerobic or facultatively anaerobic bacterium with an optimum temperature of 37 °C and a pH range of 7.2–7.4 [24]. Most strains can decompose glucose, fructose, sucrose, mannose and galactose, producing acid but not gas and can ferment mannitol but generally do not ferment lactose.

The serotypes of *P. multocida* are primarily classified according to the different characteristics of their capsule and somatic antigens [25]. Capsule antigen typing divides *P. multocida* into five capsule serotypes: A, B, D, E and F [26] (Table 1). Type A is the most common and widely distributed serotype, associated with fowl cholera, bovine respiratory disease (BRD), and pneumonia in pigs, among other hosts [27]. Type B (with arabinose, mannose and galactose as the main components of the capsule) is the primary cause of haemorrhagic septicaemia in cattle and buffaloes, and has also been occasionally reported in pigs [28]. Type D *P. multocida* (whose capsule is composed primarily of heparosan, a heparin precursor) is a major causative agent of progressive atrophic rhinitis in pigs. However, only toxigenic strains that produce the *Pasteurella multocida* toxin (PMT) are pathogenic, and type A strains, often in combination with Bordetella bronchiseptica, can also contribute to the disease [29]. Type E strains are rarely isolated and appear to be largely restricted to specific regions in Africa [30]. Although serotype E was historically associated with haemorrhagic septicaemia in cattle, subsequent studies have established that serotype B:2 is the primary etiological agent of this disease in most endemic regions, particularly in Asia [31]. Their isolation frequency varies considerably by region and host species, but they remain uncommon even within endemic areas. Type F (with chondroitin as the main component of the capsule) can be isolated from animals [32,33,34]. For somatic antigen typing, the classification of *P. multocida*’s somatic antigen (O antigen) is relatively complex. *P. multocida* can be divided into 12 serotypes through agglutination reactions, but this method has issues, such as cross-reactions and autoagglutination, which limits its application [35,36]. Another historical typing method is the Heddleston scheme, which uses a gel diffusion precipitin test to classify *P. multocida* into 16 somatic serotypes (types 1–16) based on the antigenicity of its lipopolysaccharide (LPS) [37]. However, this serological method suffers from significant limitations, including poor reproducibility, cross-reactivity, and the inability to type many isolates. Consequently, it has been largely superseded by molecular methods that assign strains to eight LPS genotypes (L1–L8) based on the genetic structure of the LPS outer core locus, which offer improved resolution and reproducibility. Isolated strains are typically designated by their capsular serotype and LPS type (e.g., A:1, D:L6) [38]. While L1, L3, L4, and L6 are the most frequently reported genotypes, primarily associated with capsular types A and D, other genotypes such as L2 (often linked to serotype B) and L5, L7, L8 have also been identified, albeit with less well-defined host and disease associations.

**Table 1:** Comparative table for *P. multocida* serotypes.

Capsular Serotype	LPS Genotype (L-Type)	Primary Hosts	Associated Diseases
A	1, 3, 4	Cattle, pigs, poultry, rabbits, cats, dogs	Pneumonia, fowl cholera, bovine respiratory disease (BRD), rabbit snuffles, wound infections (bites)
B	2, 5	Cattle, buffalo, pigs	Haemorrhagic septicaemia (in cattle/buffalo), progressive atrophic rhinitis (in pigs)
D	3, 4, 6	Pigs, cattle, rabbits	Progressive atrophic rhinitis (toxigenic strains), pneumonia
E	–	Cattle, buffalo	Rare; sporadic cases of haemorrhagic septicaemia reported mainly in Africa (note: serotype B is the primary etiological agent worldwide)
F	–	Poultry (turkeys, chickens), pigs (occasionally)	Pneumonia, fowl cholera (less common) (other L-types (e.g., L5, L7, L8) have also been reported in various isolates, but their host associations remain unclear; notably, L2 is predominantly associated with serotype B and is not typically found in serotypes A or D)

## 3. Epidemiology of *Pasteurella multocida*

*P*. *multocida* can infect a variety of animals, including domestic animals (such as pigs, cows and sheep), poultry (such as chickens, ducks and geese), wildlife and humans [39,40,41]. This wide host range makes the bacterium easily transmittable and prevalent in animal populations. In addition, *P. multocida* has diverse transmission routes, including direct contact (such as bites and scratches), indirect contact (such as contact with contaminated objects or environments), airborne transmission (such as droplet transmission) and digestive tract transmission (such as consuming contaminated food or water) [42]. These diverse transmission routes increase the risk of the bacterium spreading in animal populations and humans. The onset of *P. multocida* infection is mostly detected in young animals, and the infection has a high mortality rate [14]. The clinical manifestations of *P. multocida* infection are highly diverse and host-dependent. In rabbits and pigs, the infection can present primarily as upper respiratory tract disease, with rhinitis as a predominant symptom, or as lower respiratory tract infection leading to pneumonia. In cattle and other ungulates, *P. multocida* is a major contributor to respiratory disease complexes, such as bovine respiratory disease (BRD), often manifesting as chronic and fibrous bronchopneumonia [43,44]. Additionally, in cattle and buffalo, certain serotypes, particularly B:2, can cause acute, fatal haemorrhagic septicaemia, a systemic infection distinct from respiratory disease. In poultry, *P. multocida* subsp. *multocida* is the causative agent of fowl cholera, which often presents as an acute septicaemic disease with high mortality, rather than a localized respiratory infection. In wildlife, outbreaks frequently manifest as acute septicaemia (e.g., in saiga antelope), though chronic respiratory forms can also occur [45]. Serotypes F and D are rarely observed in poultry. In chronic or subclinical forms of fowl cholera, the respiratory tract can serve as a primary site of infection, with clinical signs including asymptomatic or mild chronic sinusitis, conjunctivitis or pneumonia; however, acute infections can progress rapidly and cause high mortality.

In livestock and wildlife, environmental and management factors play crucial roles in triggering *P. multocida* outbreaks. As an opportunistic commensal, *P. multocida* typically resides harmlessly in the upper respiratory tract; however, environmental stressors, including transportation, weaning, overcrowding, poor ventilation and dietary changes, can precipitate transition to an invasive infection [43]. Climatic factors also contribute substantially: in rabbits, elevated ammonia concentrations, suboptimal temperatures and high relative humidity (exceeding recommended 60–70%) increase pasteurellosis prevalence and severity [46,47]; in zoo animals, outbreaks have been linked to changes in rainfall, humidity and temperature [48]; and in saiga antelope, unusually high humidity and temperature preceded a mass die-off of approximately 200,000 animals from haemorrhagic septicaemia [49,50]. In addition, physiological states such as pregnancy and lactation have been associated with increased mortality in rabbits, likely due to immune modulation [47]. Collectively, these observations highlight that *P. multocida* disease emerges from a complex interplay between the pathogen, host, and environment.

*P. multocida* infections in humans primarily occur via contact with animals through bites, scratches or mucous secretions. Among these, cats and dogs are the most frequently implicated sources, reflecting their high rates of carriage and close physical proximity to humans [51]. Approximately 300,000 patients are seen annually in emergency departments in the United States alone for animal scratches or bites, and *P. multocida* is the most common pathogen associated with infections in these patients. The infection rate for cat bites (20–80%) is considerably higher than that for dog bites (3–18%) [52], and cats are involved in 60–80% of human *P. multocida* infections [53].

Following an animal bite or scratch, local symptoms progress rapidly. Common manifestations include swelling, cellulitis, and purulent exudate at the wound site [54,55]. In most cases, *P. multocida* infection remains localized to the skin and soft tissues around the bite wound. However, in severe cases—particularly in immunocompromised individuals or those with underlying comorbidities—the infection can progress to bacteraemia, sepsis, osteomyelitis, endocarditis, or meningitis [54]. The mortality rate varies considerably by infection type: it is very low for localized wound infections, approximately 8–31% for bacteraemia [56,57], and up to 30% for meningitis [58]. The risk of severe disease and death is considerably higher in immunocompromised individuals than in the general population [59,60]. The clinical outcome of *P. multocida* infection is profoundly influenced by the host’s immune status. Immunocompromised individuals, including those with malignancies, diabetes mellitus, chronic liver disease (particularly cirrhosis), end-stage renal disease or those undergoing chemotherapy or immunosuppressive therapy, are at substantially heightened risk for developing severe and invasive infections [61,62,63]. In such patients, *P. multocida* can cause fulminant infections, including bacteraemia, sepsis, endocarditis, peritonitis and septic shock, even in the absence of an overt animal bite or scratch [64,65]. Multiple risk factors have been associated with increased risk for developing septicaemia, including advanced age, chronic liver disease and diabetes mellitus. In particular, liver cirrhosis has been frequently reported in association with *P. multocida* septicaemia. Conversely, infections in immunocompetent hosts are typically milder and more localised, although severe cases have occasionally been reported [66]. These observations highlight that *P. multocida* functions as a classic opportunistic pathogen, whose pathogenic potential is magnified in the setting of compromised host defences. Furthermore, co-infections with other pathogens can predispose an individual to *P. multocida* infection. For example, SARS-CoV-2 infection has been reported to promote *P. multocida* pulmonary coinfection through alteration of the epithelial–endothelial barrier and virus-induced immunosuppression [67]. Similarly, bovine herpesvirus-1 (BHV-1) infection enhances the adherence and invasion of *P. multocida* to host cells, thus illustrating how viral co-infections can increase susceptibility to bacterial superinfection [68].

In addition to bite-related infections, *P. multocida* can also be transmitted via the respiratory route, primarily through inhalation of contaminated aerosols or droplets, or through close contact with the respiratory secretions of infected animals. Human respiratory tract infections occur frequently, mainly in patients with underlying chronic lung diseases (such as bronchiectasis or chronic obstructive pulmonary disease), manifesting as severe pneumonia, lymphadenopathy, and epiglottitis [6,69,70,71].

## 4. Virulence Factors and Pathogenesis of *Pasteurella multocida*

**Figure 2:**
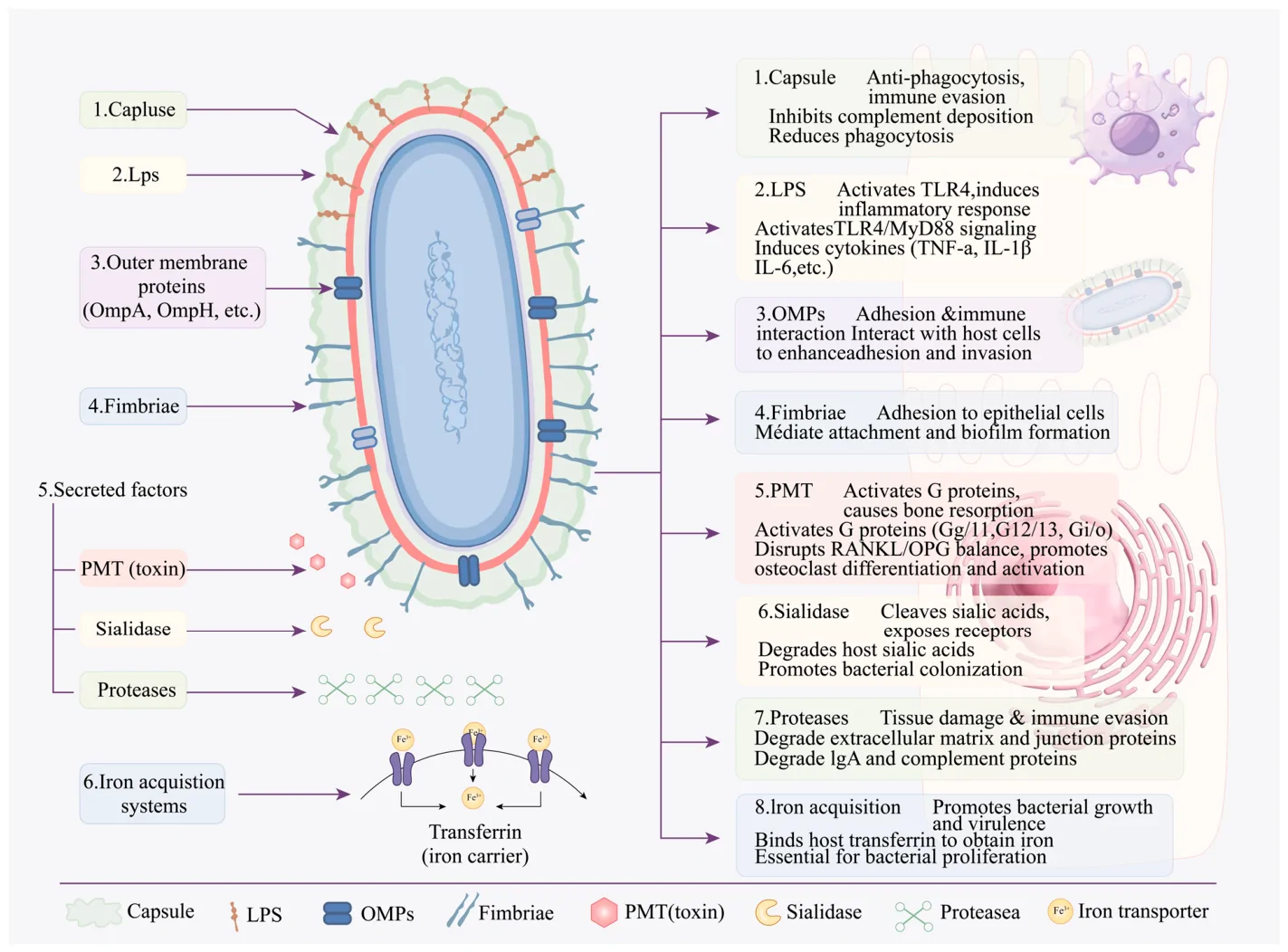
Overview of *P. multocida* virulence factors and host interaction. The arrow indicates the position of the target structure.

In addition to capsules and LPSs, the virulence factors of *P. multocida* are adhesins (PfhA and PtfA), iron acquisition systems (ExbB-TonB, HgbA and HgbB), sialidases (NanH and NanB), outer membrane protein (OMP; OmpH and Oma87) and *P. multocida* toxin (PMT) [72,73,74] (Figure 2) (Table 2). Several important virulence factors are described below. The initial step of infection requires bacterial colonization and adhesion to host cells, mediated by adhesins and fimbriae. Subsequently, the capsular polysaccharide, which mimics host glycosaminoglycans, enables the bacteria to evade innate immune recognition and phagocytosis [75,76,77]. The second obstacle is a host’s inherent immune system, which involves phagocytosis, bactericidal effect of complements and isolation of free iron. However, the structure of the pathogen’s capsular polysaccharide is similar to that of the vertebrate glycosaminoglycan and provides bacteria with conducive conditions, such as anti-phagocytosis and molecular simulation, thus enabling the bacterium to escape a host’s immune system [78]. When *P. multocida* breaks through the immune barrier and enters blood vessels, it proliferates, increases its virulence and causes bacteraemia [79]. Then, it is transported to various organs, causing tissue degeneration and necrosis, organ dysfunction, body sepsis and death. The virulence factor of *P. multocida* becomes the key factor for bacteria to invade the body and cause diseases and is the entry point for the development of vaccines and disease resistance at the immune level.

### 4.1. Capsule

*P. multocida* is a typical capsular bacterium, and the composition and structure of the capsular materials found in serotypes A, D and F are extremely similar to mammalian glycosaminoglycans, consisting mainly of hyaluronic acid (HA) [80], heparin and chondroitin. HA is a polymer of D-glucuronic acid and *N*-acetyl-D-glucosamine [81]. Glycosaminoglycan capsular polysaccharide is considered an essential virulence factor for *P. multocida*, providing protection against other surface antigens from the host’s immune system, preventing phagocytosis, and complementing bactericidal activity [82,83,84], which can cause haemorrhagic septicaemia and avian cholera [75,85]. Capsular hyaluronic acid is thought to contribute to immune evasion by mimicking host glycosaminoglycans, thereby disguising the bacterium from host immune recognition [75,82]. Al-Haddawi et al. [86] observed that *P. multocida* serotype A:3 strains adhered to and colonised lung explants, possibly because of the high affinity of HA capsular binding to specific glycoproteins in epithelial cells. Beyond its structural and functional characterisation, recent studies have revealed regulatory mechanisms governing capsule synthesis. Fis, a nucleoid-associated protein, positively regulates the transcription of capsular glycosaminoglycan genes in *P. multocida*. Fis’s expression is upregulated in vivo and correlates positively with capsular production and virulence. The deletion of Fis reduces capsular synthesis, biofilm formation, and resistance to macrophage phagocytosis [87].

### 4.2. Lipopolysaccharides

LPS is a complex molecule composed of lipid A, core oligosaccharides, and, in many Gram-negative bacteria, O-antigens. However, *P. multocida* produces a lipooligosaccharide (LOS) that lacks the highly variable O-antigen polysaccharide repeats found in enteric bacteria like E. coli or Salmonella. The structural diversity and serotypic specificity of *P. multocida* LOS are instead determined by variations in the structure of the outer core oligosaccharide, not by an O-antigen [88,89]. Lipid A is the innermost and most toxic part of LPSs. It is a scaffold of two glucosamine units connected by a β-1, 6-glucoside bond, on which multiple fatty acid chains are attached. The number and length of these fatty acid chains vary by bacterial species [90,91]. LPSs play a critical role in pathogenesis and are the major stimulators of a host’s immune response, which is a key determinant of the protective effect of bacteria [92]. LPSs induce a strong innate immune response primarily through toll-like receptor (TLR) 4, whereas other bacterial components such as lipoproteins are recognized by TLR2 [88]. Upon invasion by Gram-negative bacteria, immature dendritic cells near the site of inflammation (induced by an innate immune response) bind to LPS/LBP complexes (via CD14/MD2 receptors), triggering TLR4 activation and transforming into mature antigen-presenting cells, which then migrate to local lymph nodes. A series of antigen-specific T lymphocytes are then activated, which in turn initiates the proliferation of B cells and subsequently produces antibodies that are specific to LPSs and other bacterial components. Depending on the properties of invading bacteria, a cell-mediated immune response may be activated, and CD4 helper T cell subpopulations are recruited to the site of infection [93,94,95]. Zhao et al. [96] constructed a new attenuated live vaccine strain PMZ2 of *P. multocida*, which deleted the entire gatA gene and a part of the hptB gene that produced a truncated LPS structure. The oral or intranasal inoculation of mutant strains induces high levels of serum IgG, potent bactericidal action and considerable mucosal IgA response. In the PMZ2 oral and intranasal vaccination groups, considerable reduction in bacterial loads in the blood, spleen, liver, and lungs alleviates spleen and liver lesions. *P. multocida* LPS can induce the release of pro-inflammatory and immunomodulatory cytokines, such as interleukin (IL)-1α, IL-6, TNF-α, interferon α and IL-12 from mouse spleen cells [97]. Yap et al. [98] found that LPSs play an important role in increasing the ability of *P. multocida* B:2 to invade cells by regulating the actin microfilaments of infected cells. This type of regulation is a key step in the entry of bacteria into the bloodstream and subsequent sepsis.

### 4.3. Filamentous Hemagglutinin

*P. multocida* possesses two filamentous hemagglutinins, PfhB1 and PfhB2 [99,100]. The C-terminal contains a YopT-like cysteine protease domain. The YopT domain of PfhB2 contains a conserved Cys-His-Asp catalytic triplet that defines a member of the YopT family and has a high sequence similarity to the prototype YopT from the genus Yersinia [101]. These proteins are similar to the LspA1 and LspA2 filamentous hemagglutinins from *H. ducreyi* [102]. PfhB2 has been shown to play a key role in the pathogenesis of diseases associated with colonization or invasion of respiratory mucosal surfaces. Tatum et al. [103] found that the PfhB2 mutant of *P. multocida* was highly attenuated by nasal administration and less attenuated by intravenous administration in turkey, and the PfhB2 mutant showed no increased sensitivity to the bactericidal activity of the serum complement system. Recent studies have shown that inoculation with recombinant PfhB2 peptide P-1059 (serotype A:3) derived from *P. multocida* can protect turkeys against P-1059 attack [104].

### 4.4. OMP

Constituting the main structure of the outer membrane of Gram-negative bacteria, outer membrane proteins are related to nutrient acquisition and ion transport [72]. OmpH and OmpA are the most typical virulence factors of *P. multocida* and play an important role in bacterial infection and disease [74,105], particularly in adhesion and invasion [106]. DNA sequence analysis shows that most of the OmpA sequences of *P. multocida* can be divided into two major alleles, namely, OmpA allele (I) and allele (II). *P. multocida* strains with OmpA (I) are more aggressive and virulent than *P. multocida* strains with OmpA (II). Pm0442, which is a lipoprotein located in the outer membrane of *P. multocida* [107], was significantly upregulated in the lung tissues of mice infected with *P. multocida*, and the toxicity of Pm0442 gene deletion strain PmCQ2Δ0442 was significantly reduced [108]. After OmpH gene deletion, bacterial adhesion ability and virulence were significantly reduced [109]. Recombinant PmOmpA induced a strong Th2 type immune response in mice and produced high immunoglobulin G1 antibodies [110]. Okay et al. [111] demonstrated the immunogenicity and protective efficacy of recombinant lipoprotein E and OmpH of *P. multocida* A:3 in mice.

### 4.5. Iron Carrier

Iron is an essential growth factor for almost all bacteria, and low concentrations of free iron on mucous membranes and tissues are a host’s first lines of defence against bacterial infection; access to iron may be a major determining factor for pathogens to maintain themselves in animal hosts [112]. Iron regulatory outer membrane proteins belong to a class of proteins embedded in the outer membrane of *P. multocida* and can participate in bacterial adhesion, invasion and immune escape, thus affecting virulence [113,114,115]. *P. multocida* produces siderophores and non-classical transferrin receptors that promote iron absorption [116,117]. Haemoglobin-binding receptors have been characterized [118,119]. Luo et al. [120] expressed and purified aspartate amino lyase (AspA), glycerol diester kinase (DgK) and 30S ribosomal protein S6 (RpsF) to characterise three novel proteins identified under iron-restricted conditions and used them to immunise chickens; the results showed that AspA, DgK and RpsF proteins induced 80.0%, 66.7% and 80.0% immunity, respectively. These data suggest that the three novel proteins identified in the supernatant of the medium play an important role in the survival of bacteria under iron-restricted conditions and thereby protect chickens from *Pasteurella multocida.* Kharb et al. [121] showed that the extraction of the outer membrane proteins of *P. multocida* B:2 cultured under normal conditions (OMP) and iron deficiency conditions (IROMP) induced a higher antibody response. Notably, Fis also determines whether *P. multocida* can utilise bound iron ions for survival, as Fis deletion severely restricts iron acquisition and attenuates pathogenicity [87]. This expands our understanding of iron homeostasis beyond conventional iron carriers.

### 4.6. Other Virulence Factors

*P. multocida* toxin (PMT) is another important virulence factor. It is a 146 kDa heat-labile toxin encoded by the *toxA* gene and is the primary causative agent of progressive atrophic rhinitis (PAR) in pigs [122]. PMT activates at least four G-protein families: Gq/11, G12/13, Gi/o, and Gs. Through its deamidase activity, PMT irreversibly activates Gq and Gα13, leading to RhoA inactivation in osteoblasts and subsequent suppression of bone formation. Concurrently, PMT upregulates RANKL expression, which promotes osteoclast differentiation and activity [123,124]. The net effect of these combined actions—reduced bone formation coupled with increased bone resorption—ultimately leads to turbinate atrophy [125,126]. Kim et al. [127] demonstrated that deletion of the toxA gene significantly reduced the toxicity of *P. multocida*, confirming the essential role of PMT in the toxigenesis of this bacterium. *P. multocida* uses sialases to hydrolyse sialic acid and obtain nutrients and has two genes encoding sialases: NanH and NanB. NanH tends to hydrolyse Neu5Acα2-3Gal, which is normally present in bird cells, whereas NanB, which is a broad-spectrum sialase, tends to hydrolyse Neu5Acα2–6Gal [128].

In addition to the well-characterised factors discussed above, recent epidemiological studies have systematically screened the prevalence of multiple virulence-associated genes in clinical isolates. A survey of respiratory cases revealed that plpB, tadD, gatG and hgbA were commonly detected in both serogroup B and E strains, whereas ompA, toxA, pcgD, latB, nctB, ppgB, natG, hgbB and exbB were absent in all isolates [129]. Notably, although serogroup B isolates carried fewer virulence genes, an inactivated vaccine derived from them induced a strong immune response in mice. However, it failed to provide cross-protection against the local serogroup E strain. This observation suggests that capsular serotype and the presence of individual virulence genes do not always correlate with cross-protective immunity, thereby emphasising the complexity of vaccine development against heterogeneous *P. multocida* populations.

**Table 2:** Major virulence factors of *Pasteurella multocida*, their biological functions, host targets, and associated diseases.

Virulence Factor	Category	Biological Function(s)	Host Target(s)/Mechanism	Associated Diseases/Outcomes
Capsule	Polysaccharide	Anti-phagocytosis, resistance to serum complement, biofilm formation	Physical barrier; inhibits macrophage uptake	Systemic infection, sepsis, pneumonia
LPS	Endotoxin	Induces pro-inflammatory cytokines (TNF-α, IL-1β, IL-6); activates TLR4/MD-2 complex	TLR4 on macrophages; induces endotoxic shock	Endotoxic shock, inflammation, tissue damage
Adhesins	Surface proteins	Adhesion to host epithelial cells	Respiratory epithelial cells; promotes colonisation	Respiratory tract colonisation, pneumonia
Fimbriae (pili)	Surface appendages	Mediate adherence and biofilm formation	Host–cell surface receptors; mediates initial attachment	Persistence, biofilm-associated infections
OMPs (OmpA, OmpH, Plp)	Surface proteins	Adhesion, invasion, nutrient acquisition, immune evasion	Epithelial cells; binds fibronectin and other extracellular matrix components	Respiratory infection, systemic spread
Iron acquisition systems	Transport proteins	Scavenging iron from host transferrin/lactoferrin via Tf receptors, heme receptors, and siderophores	Transferrin, lactoferrin in host serum	In vivo growth, systemic infection
Sialidase (neuraminidase)	Enzyme	Cleaves host sialic acid, promotes colonisation	Mucosal glycoproteins; enhances biofilm	Respiratory tract colonisation
PMT (toxin)	Protein toxin	Activates G-protein families (Gq/11, G12/13, Gi/o and Gs); stimulates osteoclast formation via deamidation of Gα subunits	Osteoblasts, osteoclasts; Rho GTPase activation	Progressive atrophic rhinitis (PAR) in pigs; bone atrophy
Proteases	Enzymes	Degrades host immunoglobulins and complement components	Immunoglobulins, complement proteins	Immune evasion, tissue damage

## 5. *Pasteurella multocida* Infection Induces Inflammasome Activation

### 5.1. Pasteurella multocida and Inflammasomes

Inflammasomes are multi-protein complexes formed in cells and are activated in response to sensing pathogens or danger signals within the cells, leading to inflammatory responses specifically associated with the initiation of a form of cell death called pyroptosis [130]. Inflammasomes belong to the nucleotide-binding oligomerising domains (NOD)-like receptor (NLR) family and play an important role in innate immunity against microbial infections. To date, many protein receptors have been shown to assemble inflammasomes, such as NLR family members NLRP1, NLRP3, NLRP6, NLRP7, NLRC4, as well as AIM2 (absent in melanoma 2) [131]. In response to microbial infection, the host innate immune system is activated by the interaction of immune cells and pathogens. Initiation of innate immune responses depends on activation of pattern recognition receptors (PRRs), including TLRs and NLRs that sense PAMPs and DAMPs [132,133].

The pulmonary response after *P. multocida* infection mainly includes the expression of some inflammatory mediators and apoptosis [134]. For example, the inoculation of *P. multocida* through transduction induces the mRNA expression of pro-inflammatory cytokines such as TNF-α, IL-6, IL-1, IL-8 and IL-12, and increases the number of bovine lung neutrophils [135]. RNA-seq analysis was used to comprehensively explore the gene expression profile of the highly virulent *P. multocida* serotype A strain CQ2 (PmCQ2) in the lungs of infected and uninfected mice. Transcriptome sequencing results showed considerable changes in the lung expression profile during in vivo *P. multocida* infection. Most of the altered genes were associated with pattern recognition receptors (PRRs) and the downstream signalling of PRRs and cytokines, suggesting a complex immune response in the lungs during *P. multocida* infection [136]. Further transcriptomic analysis of the host response induced by PmCQ2 (*P. multocida* strain CQ2, serotype A) revealed differential expression of genes encoding putative virulence factors and components of the NLR signalling pathway [137,138]. These findings suggest that multiple virulence factors may contribute to infection and that NLR signalling might be activated following PmCQ2 infection. However, these transcriptomic observations require validation at the protein level and in functional studies.

While the above studies collectively establish that *P. multocida* infection triggers NLR signalling and inflammasome activation, several fundamental questions remain unresolved. First, most of the current evidence relies on transcriptomic profiling and in vitro macrophage infection models, with limited validation at the protein level or in physiologically relevant in vivo systems. Second, the specific pathogen-associated molecular pattern molecules (PAMPs) of *P. multocida* that are recognised by NLRP3 or NLRP6 have not been definitively identified. Whether lipopolysaccharide (LPS), capsule components or secreted toxins serve as the primary trigger remains speculative. Third, the cell-type specificity of inflammasome activation has not been systematically examined; it is unclear whether epithelial cells, alveolar macrophages or neutrophils are the primary responders during respiratory infection. These gaps underscore the need for future studies combining genetic knockout models, biochemical ligand–receptor characterisation, and spatial transcriptomics to delineate the spatiotemporal dynamics of inflammasome activation during *P. multocida* infection.

Host genetic factors are increasingly recognised as determinants of susceptibility to *P. multocida* infection. Comparative genomic analyses have revealed host-correlated phylogenetic clustering, with strains from cattle, pigs, poultry and cats forming distinct clades, and multi-locus sequence typing has identified strong host–sequence type associations (e.g., ST1 in cattle, ST11/ST10/ST3 in pigs and ST30 in cats) [25,26]. However, systematic studies on host genetic polymorphisms (e.g., in TLR, NLR or cytokine genes) remain scarce. In poultry, breed-specific differences in resistance have been documented: indigenous Ri chickens were more susceptible to *P. multocida* than commercial Luong Phuong chickens, with one MHC haplotype associated with pathological lesions and mortality [139]. Genetic variation in resistance has also been observed in turkeys [140]. These underlying genetic variants have not been definitively identified, representing a substantial knowledge gap that could inform selective breeding for disease-resistant livestock and the identification of high-risk human populations.

### 5.2. Pasteurella multocida Can Activate the NLRP3/NLRP6 Inflammasome

**Figure 3:**
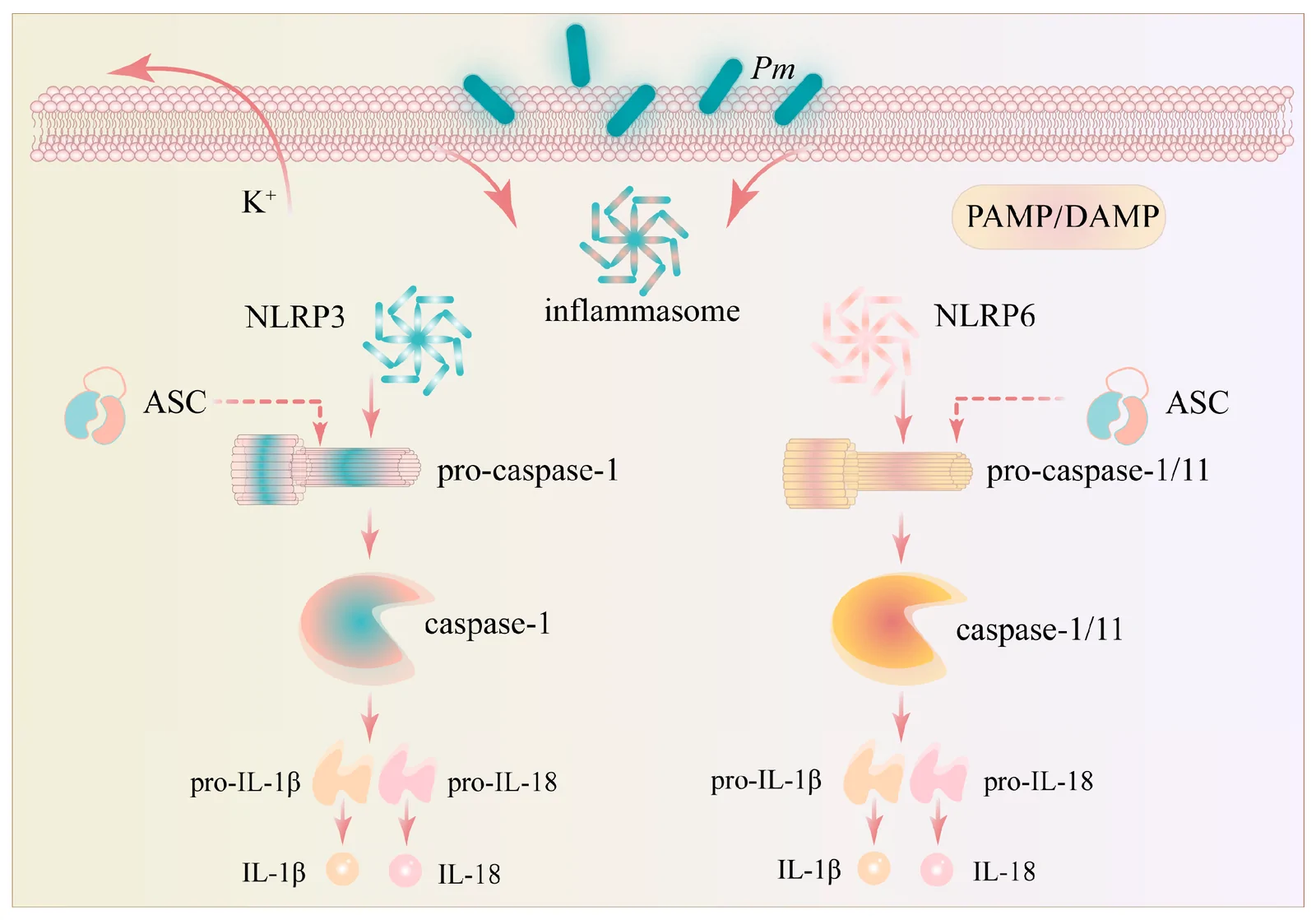
Mechanism of activation of NLRP3/NLRP6 inflammasome by *Pasteurella multocida*. The arrows indicate the direction of progression to the subsequent step/process. Different colors are used solely for visual distinction between the components and have no specific scientific meaning.

NLRP3 is a key protein in the inflammasome complex and is a member of the NLR family. It is the core component of inflammasomes and is one of the important PRRs during *P. multocida* infection. When NLRP3 is activated, ASC acts as a connector protein, connecting NLRP3 to downstream effector molecules. ASC then recruits and activates caspase-1, which is a cysteine protease that can shear and activate inflammatory cytokines, such as IL-1β and IL-18 [141]. After NLRP3 inflammasome assembly, the dormant procaspase-1 protein is hydrolysed and cleaved to active caspase-1 [142]. Subsequently, caspase-1 converts the cytokine precursors pro-IL-1β and pro-IL-18 into mature and biologically active IL-1β and IL-18 and induces a type of pro-inflammatory cell death called pyroptosis [143,144]. The role of NLRP3 inflammasome in response to microbial infection has been extensively studied. The NLRP6 inflammasome is a relatively new member of the NLR family. Similar to NLRP3 activation, NLRP6 activation triggered by PAMPs or DAMPs induces the recruitment of caspase-1 via the adaptor ASC, leading to an inflammatory response and the secretion of IL-1β and IL-18 [145,146] (Figure 3).

Several studies have provided evidence for the involvement of the NLRP3 inflammasome in *P. multocida* infection. Fang et al. [147] reported that NLRP3 inflammasome activation could be induced in cattle following PmCQ2 infection, thus providing one of the first in vivo observations in a natural host species. In a subsequent mechanistic study using macrophage cell lines, Ran et al. [148] found that the scaffold protein RACK1 was positively regulated with PmCQ2-induced NLRP3 activation; specifically, siRNA RACK1 knockdown attenuated caspase-1 activation and IL-1β production, whereas RACK1 overexpression enhanced these responses. While these loss- and gain-of-function experiments strongly suggest a regulatory role for RACK1, the findings are currently limited to immortalised cell lines and require confirmation in primary cells or in vivo.

A notable and counterintuitive observation was reported by Fang et al. [149], who compared NLRP3 inflammasome activation induced by high (PmCQ2) and low (PmCQ6) virulence strains. Surprisingly, the low-virulence strain induced higher levels of NLRP3 transcription, caspase-1 activation and mature IL-1β secretion than the highly virulent isolate. The authors attributed this difference to the thicker capsule of PmCQ2, which suggests that capsule thickness may influence bacterial colonisation and modulate NLRP3 activation. However, this interpretation remains correlative, and direct causal evidence linking capsule thickness to inflammasome modulation is currently lacking. Furthermore, it is worth noting that the identity of the specific *P. multocida* component(s) recognised by NLRP3 has not been established. In addition, Fang et al. [147] observed that NLRP3-deficient macrophages exhibited significantly reduced caspase-1 activation and IL-1β secretion in response to *P. multocida* and suggested that splenic tyrosine kinase may be involved in this process, although the underlying mechanism remains speculative.

Regarding the NLRP6 inflammasome, Wu et al. [146] investigated its role using an in vivo mouse model. They found that *P. multocida* induced severe pulmonary inflammation with extensive immune cell infiltration in both wild-type and Nlrp6-deficient mice. Interestingly, Nlrp6-deficient mice were more susceptible to infection, exhibiting higher bacterial loads and reduced numbers of immune cells in the lungs. The authors proposed that NLRP6 mediates caspase-1 activation and ASC oligomerisation, which leads to IL-1β secretion, and also regulates the expression of chemokines (CXCL1, CXCL2, and CXCR2) that promote neutrophil recruitment. Based on these observations, Wu et al. [146] proposed a possible synergistic effect between NLRP6 and NLRP3 during *P. multocida* infection. However, this hypothesis is currently based on single-gene knockout studies and requires formal validation using Nlrp3/Nlrp6 double-knockout models to confirm the interplay and relative contributions of each inflammasome pathway.

Although the involvement of NLRP3 and NLRP6 in *P. multocida* infection is supported by multiple studies, several inconsistencies and knowledge gaps warrant further attention. First, the relationship between bacterial virulence and inflammasome activation appears paradoxical. Fang et al. [149] reported that a low-virulence strain (PmCQ6) induced stronger NLRP3 activation than a highly virulent strain (PmCQ2), which suggests that robust inflammasome responses do not necessarily correlate with bacterial pathogenicity. This raises the important question of whether inflammasome activation is primarily a host protective response or, conversely, a pathological driver of tissue damage. Second, the interplay between NLRP3 and NLRP6 remains poorly understood. Wu et al. [146] suggested a possible synergistic effect, but the molecular basis of this synergy, whether through shared downstream signalling, reciprocal regulation or independent parallel pathways, has not been elucidated. Third, most studies have focused on acute infection models, leaving the role of inflammasomes in chronic or persistent *P. multocida* infections unexplored. In addition, the translational relevance of these findings to human infections and livestock disease management has not been adequately addressed. Future research should prioritise the use of cell-type-specific knockout models and longitudinal in vivo studies to resolve these uncertainties.

While our review focuses on the well-supported roles of NLRP3 and NLRP6, it remains to be determined whether other inflammasome sensors are also activated during *P. multocida* infection. Notably, recent studies have demonstrated that L-serine [145] and glycine [150] can reduce the production of inflammatory cytokines in macrophages by blocking the activation of multiple inflammasome pathways, including NLRP1, NLRP3, NLRC4, AIM2, and caspase-1, in PmCQ2-infected macrophages. These pharmacological observations raise the possibility that NLRC4 and AIM2 could participate in host defence against *P. multocida* infection, which still awaits validation via gene-deficient models.

### 5.3. K^+^ Outflow and Nek7 Play a Key Role in the Activation of NLRP3 Inflammasome Induced by Pasteurella multocida

K^+^ efflux has been identified as a critical upstream event for NLRP3 inflammasome activation in various infection models [151]. In the context of *P. multocida*, emerging evidence suggests that K^+^ outflow may similarly contribute to NLRP3-dependent pyroptosis. It has been proposed that *P. multocida* infection may trigger K^+^ efflux by disrupting potassium channels in the cell membrane or interfering with potassium transport systems. However, the precise molecular mechanism remains poorly elucidated. Furthermore, K^+^-efflux-mediated NLRP3 inflammasome activation requires Nek7, a member of the mammalian NIMA-associated kinase family, which has been reported to interact directly with NLRP3 [151,152].

Wang et al. [153] investigated the role of K^+^ efflux and Nek7 in PmCQ2-induced inflammasome activation using macrophage cell lines. They demonstrated that K^+^ efflux mediates PmCQ2-induced IL-1β secretion and that pharmacological blockade of K^+^ efflux attenuates caspase-1 activation and ASC oligomerisation. In addition, they observed that Nek7 is involved in PmCQ2-induced caspase-1 activation and IL-1β secretion, and that PmCQ2 infection promotes Nek7–NLRP3 interaction in a K^+^ efflux-dependent manner.

This demonstration that K^+^ efflux and Nek7 are essential for NLRP3 inflammasome activation by *P. multocida* represents an important mechanistic advance [151,152,153]. However, several critical questions remain. First, the upstream signal that triggers K^+^ efflux following *P. multocida* infection has not been identified. This includes whether it is mediated by a specific bacterial toxin, pore-forming protein or host-derived damage signals. Second, it is unclear whether Nek7 is exclusively required for NLRP3 activation induced by *P. multocida*, or whether it also participates in other inflammasome pathways (e.g., NLRP6 or NLRC4) during infection. Third, the physiological relevance of the Nek7–NLRP3 interaction needs to be validated in primary cells and in vivo models, as current evidence is predominantly derived from immortalised macrophage cell lines. Fourth, the temporal relationship between K^+^ efflux, Nek7 recruitment and ASC oligomerisation has not been resolved with sufficient kinetic resolution. Addressing these questions will be essential for translating these mechanistic insights into therapeutic strategies for *P. multocida*-associated diseases.

### 5.4. Knowledge Gaps and Future Directions in Inflammasome Research During P. multocida Infection

Despite the progress summarised above, the field currently suffers from several interconnected limitations that constrain the fundamental understanding and clinical translation: (i) Species- and strain-specific differences: Most mechanistic studies have been performed with a limited number of *P. multocida* strains (primarily PmCQ2 and PmCQ6) in murine or bovine models. Whether the observed inflammasome activation patterns are generalisable across different capsular serotypes (A, B and D) and host species remains unknown. (ii) Conflicting observations on bacterial virulence: As noted above, the observation that low-virulence strains may elicit stronger inflammasome responses than highly virulent ones challenges the assumption that inflammasome activation is a correlate of virulence. This paradox may be explained by differences in capsule thickness [149], potentially combined with variations in LPS structure or the expression of immunomodulatory virulence factors that actively suppress host sensing—although these latter possibilities remain speculative. This area warrants additional systematic comparative studies. (iii) Signalling cross-talk and redundancy: The potential interplay between NLRP3, NLRP6 and other inflammasome sensors (e.g., NLRP1, NLRC4) during *P. multocida* infection has not been explored. Multiple sensors may operate hierarchically or redundantly, depending on the infection stage or bacterial load. Multi-gene knockout approaches are required to dissect this complexity. (iv) Translational gap: While animal models have provided valuable insights, the relevance of these findings to human *P. multocida* infections, particularly in immunocompromised patients, the elderly and those with chronic lung diseases, remains largely unexamined. Furthermore, the potential for targeting inflammasome components as therapeutic interventions in veterinary medicine has not been sufficiently investigated.

Addressing these knowledge gaps will require a concerted effort involving standardised infection models, multi-omics approaches and collaborative research bridging basic immunology and veterinary clinical practice.

## 6. Conclusions and Prospects

A study of the virulence factors of *P. multocida* is helpful to the examination of its pathogenic mechanism to a host and the development of new vaccines. The in-depth analysis of pathogenic and immune mechanisms will provide insight into the molecular basis of host–microbe interactions in chronic infections and the transition from subclinical or chronic diseases to acute transmissible diseases. In this review, the pathogenic mechanism of *P. multocida* and its relationship with the activation of inflammasomes were discussed, and the complex mechanism of the pathogenic bacteria in causing diseases in livestock and humans was revealed. The pathogenesis of *P. multocida* and the activation of inflammasomes still have many directions worth exploring. First, the interaction between the various virulence factors of *P. multocida* and its specific contribution to the pathogenesis process needs to be further clarified. This information is essential to the development of novel drugs or vaccines targeting specific virulence factors. Second, the mechanism of inflammasome activation in anti-infection immunity still needs to be further studied, especially the activation mode of inflammasomes and its influence on disease outcomes in different infection stages and host contexts. In addition, modern biotechnological means, such as high-throughput sequencing, proteomics and metabolomics, will be used to fully analyse host–pathogen interactions during *P. multocida* infection, and identify new perspectives and strategies for disease prevention and control.

## Data Availability

No new data were created or analyzed in this study. Data sharing is not applicable to this article.
